# Do Event-Related Evoked Potentials Reflect Apathy Tendency and Motivation?

**DOI:** 10.3389/fnhum.2018.00011

**Published:** 2018-01-31

**Authors:** Hiroyuki Takayoshi, Keiichi Onoda, Shuhei Yamaguchi

**Affiliations:** Department of Neurology, Shimane University, Izumo, Japan

**Keywords:** motivation, apathy, event-related potential, P2, P3

## Abstract

Apathy is a mental state of diminished motivation. Although the reward system as the foundation of the motivation in the human brain has been studied extensively with neuroimaging techniques, the electrophysiological correlates of motivation and apathy have not been fully explored. Thus, in 14 healthy volunteers, we examined whether event-related evoked potentials (ERP) obtained during a simple number discrimination task with/without rewards reflected apathy tendency and a reward-dependent tendency, which were assessed separately using the apathy scale and the temperament and character inventory (TCI). Participants were asked to judge the size of a number, and received feedback based on their performance in each trial. The P3 amplitudes related to the feedback stimuli increased only in the reward condition. Furthermore, the P2 amplitudes related to the negative feedback stimuli in the reward condition had a positive correlation with the reward-dependent tendency in TCI, whereas the P3 amplitudes related to the positive feedback stimuli had a negative correlation with the apathy score. Our result suggests that the P2 and P3 ERPs to reward-related feedback stimuli are modulated in a distinctive manner by the motivational reward dependence and apathy tendency, and thus the current paradigm may be useful for investigating the brain activity associated with motivation.

## Introduction

Apathy is one of the representative clinical symptoms with reduced motivation. Apathy is defined as diminished motivation that is not attributable to a disorder of consciousness, cognitive impairment, or emotional distress (Marin, 1990), and it is characterized by an absence of will which results in decreased self-initiated behavior (Berrios and Grli, 1995). It is difficult to judge clinically whether there is an absence of will or not, but Stuss et al. defined apathy as a status characterized by a decreased response to external stimuli (Stuss et al., 2000). Apathy is seen frequently in various neuropsychiatric disorders, but its mechanism has not been fully explored. If apathy can be assessed by physiological measures, the exploration of neural basis of motivation may be understood more deeply.

Electrophysiological indices such as the P3 event-related evoked potential (ERP), feedback-related-negativity (FRN), and stimulus preceding negativity (SPN) are objective measures of cognitive processing, which have excellent temporal resolution for neural activities elicited by external and internal events. Several ERP studies have examined the motivational changes caused by monetary gain or loss, and it is known that some ERP components are particularly sensitive to valence and the size of a reward. One of these components is P3 and increases in its amplitude are associated with the gain of a larger reward (Yeung and Sanfey, 2004; Sato et al., 2005). However, few studies have examined the direct relationship between P3 and apathy. In particular, the P3 amplitude in a visual oddball task decreased in apathetic patients after stroke (Yamagata et al., 2004). A similar result was found in Parkinson's disease based on a visual oddball task (Kaufman et al., 2016). The P3 amplitude also decreased in patients with anhedonia (Dubal et al., 2000) and depression (Foti and Hajcak, 2009). This evidence suggests that P3 may reflect cognitive processes that are sensitive to an apathetic state.

Another component is the FRN, which was discovered as a negative potential generated by feedback stimuli signifying a false response (Takasawa et al., 1990). The FRN was also elicited by feedback signifying monetary loss (Gehring and Willoughby, 2002). The FRN amplitude was higher when immediately preceding feedback represented monetary gain compared with loss (Masaki et al., 2006), thereby indicating that the FRN is affected by the motivation level in a trial base. The FRN is generated in the anterior cingulate cortex (ACC), and dysfunction of the ACC network is associated with apathy (Onoda and Yamaguchi, 2015). This evidence suggests that the FRN might also reflect the degree of apathy.

In addition, the SPN was associated with reward gain in motivation studies, including a task with feedback signals related to performance (Brunia and Damen, 1988). The SPN was studied in a time production task, and it had a larger amplitude in the case with monetary rewards (Bocker et al., 1994). Therefore, it is possible that the SPN also reflects motivation.

The ERP component is known to correlate with personality traits and affective disorder (Gangadhar et al., 1993; Hansenne, 1999). To make a physiological index of apathy, the effect of other motivation-related factors should be considered simultaneously. Here, we focused on reward dependence, novelty seeking, and depression. Reward dependence is characterized by eager to help and please others, persistent, industrious, warmly sympathetic, sentimental, and sensitive to social cues and personal succor but able to delay gratification with the expectation of eventually being rewarded (Cloninger, 1987). These characteristics suggest that reward dependence could be treated as a motivational trait. Novelty seeking is a temperament associated with exploratory activity in response to novel stimulation, impulsive decision making, extravagance in approach to reward cues, quick loss of temper, and avoidance of frustration (Cloninger et al., 1993). Reward dependence and novelty seeking are related with reward system (Krebs et al., 2009). Moreover, novelty seeking is associated with dopamine function (Cloninger et al., 1994) and its polymorphism (Lusher et al., 2001). Novelty seeking may play a role in motivation. On the other hand, depression is associated with anhedonia and loss of motivation through functional impairment of the mesolimbic dopamine pathway (Martin-Soelch, 2009). Apathy and depression are often confused, and sometimes both could be seen simultaneously, particularly in neurological disorders (Hama et al., 2011). It would be desirable to distinguish apathy and depression to reveal the neural basis. Therefore, we investigated the relationships between the ERPs and not only apathy but also reward dependence, novelty seeking, and depression.

In this study, we developed a new simple task where the P3, FRN, and SPN components were evaluated in a single session, and motivation was modulated by changing a monetary reward. This task paradigm enabled us to examine the relationships among the electrophysiological measures, novelty seeking, reward dependence, depressive state, and apathy tendency.

## Methods

### Subjects

Fourteen neurologically healthy adult volunteers (eight males, six females) were recruited. Their mean age was 25.3 years (standard deviation = 4.1, range = 20–35 years). All subjects had normal vision or corrected to normal vision. This study was approved by the Ethics Committee of Shimane University, and was conducted in accordance with the Declaration of Helsinki.

### Questionnaires

Participants completed the apathy scale (Starr et al., 1983; Okada et al., 1998), the temperament and character inventory (TCI) (Cloninger et al., 1993; Kijima et al., 1996), and Zung's self-rating depression scale (Zung, 1965). These questionnaires are self-entry style questionnaires. TCI is a 125-item questionnaire regarding personality developed by Cloninger et al. (4 points scale per item). We obtained scores for reward dependence and novelty seeking because both traits are closely related to motivation. A higher score of novelty seeking represents novelty seekers (Cloninger et al., 1993). A higher score of reward dependence represents more motivated state (Kijima et al., 1996). A higher scores of apathy scale and SDS represent more apathetic state and depressive state, respectively. The score of mean and standard deviation for novelty seeking was 47.1 ± 6.9, for reward dependence was 41.8 ± 4.5(33–49), for harm avoidance was 51.6 ± 5.9(41–63), for apathy score was 11.3 ± 5.5(2–21), and for SDS was 36.4 ± 8.4(20–52). There were several correlations among apathy scale, SDS, and temperaments. Apathy scale was positively correlated with harm avoidance and SDS (Supplementary Table 1).

### Procedures

**Figure 1:**
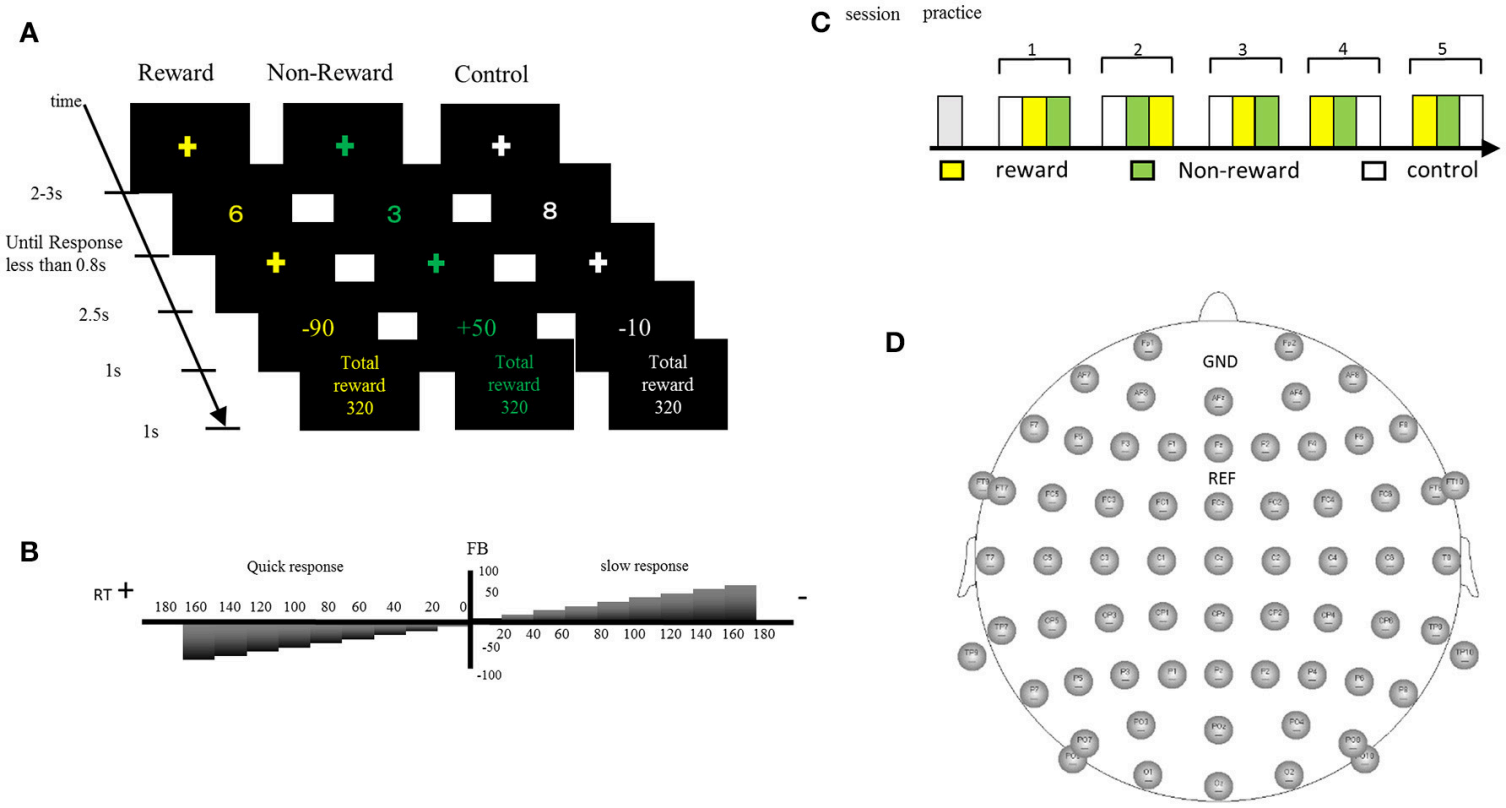
**(A)** In the number discrimination task, participants pressed the assigned button after judging the size of the number as more than five or less. A monetary reward (reward condition) or reaction speed (non-reward condition) was presented as a feedback stimulus. In the control condition, the feedback was a random number. **(B)** The feedback value increased when the button was pressed faster than a criterion, whereas it became smaller when the button was pressed slower than the criterion. **(C)** Sequence of the stimulus sessions. The task comprised five sessions and each session included three blocks. Participants practiced 20 trials before starting the task. **(D)** Electrode positions.

We developed an original task to measure SPN, FRN, and P3 in a single experimental session. Participants were asked to perform a number discrimination task. Figure 1 shows the protocol for the number discrimination task. This task comprised three conditions (reward, non-reward, and control condition). In each trial, a number excluding five was displayed and participants judged whether the number is smaller than five. Participants were asked to press the left button when the number was smaller than five and to press the right button when the number was larger than five. The feedback stimulus was presented 2.5 s after the response. When participants correctly responded faster than the criterion time, a positive feedback stimulus was presented with a value of +10 to +90 at an interval of 10. In contrast, when they responded correctly but slower than the criterion time, a negative feedback was presented with a value of −10 to −90 at an interval of 10. The feedback value was altered based on the response speed and accuracy in the trial, which faster responses yielded higher values, and vice versa (see Figure 1B for details). If the previous response was correct and faster than the criterion, the next criterion was shortened automatically by 10 ms. Inversely, if the previous response was incorrect or too slow, the following criterion was automatically prolonged by 10 ms. In the case of the reward condition, the positive value was represented by a monetary reward and it was added to the total amount of money acquired. Even if the feedback was negative, the total amount of money acquired was not decreased because the expected total reward was manipulated to be positive in the reward condition. In the non-reward condition, the value of the feedback stimulus indicated the response speed, which did not affect the acquisition of money. In the case of the control condition, the value of the feedback stimulus ranging from +90 to −90 at an interval of 10 was assigned randomly regardless of the response speed. The probabilities of positive and negative feedback were manipulated so they were both kept at 50%. When participants made a wrong response, “incorrect response” was presented as text in all conditions. If no response was made for 0.8 s after the presentation of the number, “no response” was presented as text. The duration of the feedback stimulus was 1.0 s. After feedback, the current total monetary reward was displayed for 1.0 s. The stimulus color differed in each condition (reward: yellow; non-reward: green; control: white). The average time of the inter-trial interval was 2.5 s (range: 2.0–3.0 s). The task comprised five sessions and each session included three blocks (one block per condition; Figure 1C). Each block included 20 trials. A break for a few minutes was allowed between the sessions. Participants were given an opportunity to practice 20 trials before the actual task. They were instructed to press a button as quickly as possible. The initial time criterion was calculated based on the mean reaction time for correct responses in the practice section for each participant. Participants were told that the positive feedback value would be larger if they pressed the button as quickly as possible and answered correctly, and that the negative feedback value would be larger if they responded slowly even with a correct response. We also told the participants that they could identify the ongoing condition based on the stimulus color.

### ERP data acquisition and signal processing

Participants were seated ~1 m from a monitor in a shielded room. Electroencephalographic (EEG) data were acquired using a BrainAmp system with 64-channel electrodes (Brain Products, Brain AMP DC, Germany) (Figure 1D). EEG signals were recorded continuously with the bandpass set at 0.01–250 Hz and a sampling frequency of 500 Hz. The reference channel was Cz, and re-referencing was performed offline based on the average of all recording sites. Noise components including ocular movement were removed by independent component analysis. The continuous EEG was segmented into epochs, including 200 ms pre-stimulus and 800 ms post-stimulus for the target, and feedback stimulus with a bandpass filter of 2–16 Hz to analyze the P2, P3, and FRN components. This filter setting was used to detect more prominent FRN and to remove slow drift with low frequency filter (Onoda et al., 2010). P2 and P3 were identified as positive or negative components in latency windows of 100–250, 200–350, and 300–500 ms, respectively. FRN was measured as a negative component in the latency window of 250–400 ms for the subtraction waveform (negative-positive). The peak amplitude and latency for each component were determined in the same window. To analyze the SPN, epochs from 2,000 ms pre-stimulus to 500 ms post-stimulus were extracted from the EEG with a bandpass filter of 0.016–30 Hz. The baseline for the SPN was defined as the time window from −1,500 to −1,000 ms before the feedback stimulus. The mean amplitude of the SPN was measured between 1000 ms pre-stimulus and stimulus onset.

### Statistics

Behavioral measures were subjected to repeated one-way analysis of variance (ANOVA) with the condition. The primary analysis models for the amplitude and latency of the ERP components comprised repeated measures ANOVA with two or three factors (channel × condition, or channel × condition × feedback valence). The Greenhouse–Geisser correction was applied to ANOVA. In the *post-hoc* test, the Bonferroni method was employed for multiple comparisons. The statistical significance threshold was set to *p* < 0.05.

## Results

According to the behavioral data, the main effect of condition on the reaction time was significant [*F*_(2, 26)_ = 7.70, ε = 0.68, *p* = 0.008, d = 0.37], where the reaction time to targets was faster for the reward condition compared with the non-reward and control conditions (*p*s < 0.05, Table 1). There was no significant main effect on the error rate [*F*_(2, 26)_ = 0.72, n.s.]. The mean total monetary reward was 1912 ± 698 yen.

**Table 1:** Response time and error rate in rewarded discrimination task.

	**Reward**	**Non-reward**	**Control**	**Statistic**
RT(ms)	372 ± 35	388 ± 28	395 ± 28	R<N, C
Error rate	2.2%	1.9%	1.75%	n.s.

*R, reward; N, non-reward; C, control P < 0.05; Statistics, repeated ANNOVA*.

**Figure 2:**
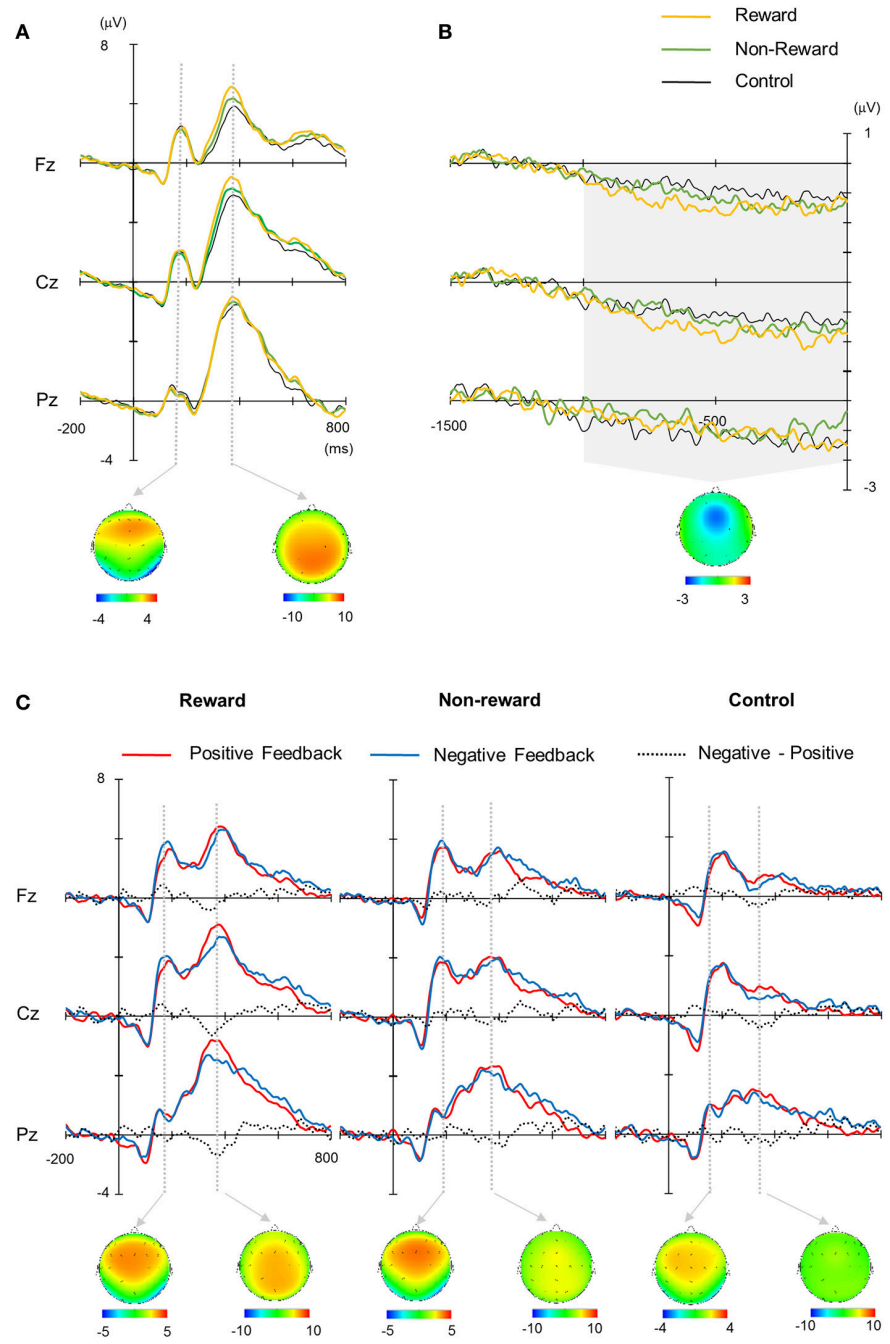
**(A)** Grand average waveforms for target stimuli. **(B)** Stimulus preceding negativity (SPN). **(C)** Grand average waveforms for feedback stimuli. Each topography was obtained across conditions (mean average of the reward, non-reward, and control conditions for target stimuli **(A)**, positive and negative conditions for feedback stimuli **(C)** at the peak of the grand average waveform. The latency for depicting each topography was 188 ms for target P2, 372 ms for target P3, 188 ms for feedback P2, and 364 ms for feedback P3. The topography of SPN was made from the mean amplitude between 1,000 ms pre-stimulus and stimulus onset **(B)**.

The grand average waveforms are illustrated in Figure 2. P2 and P3 were elicited for both the target and feedback stimuli, and SPN appeared to precede the feedback stimuli. These components differed in their amplitude and latency depending on the condition or feedback valence.

**Figure 3:**
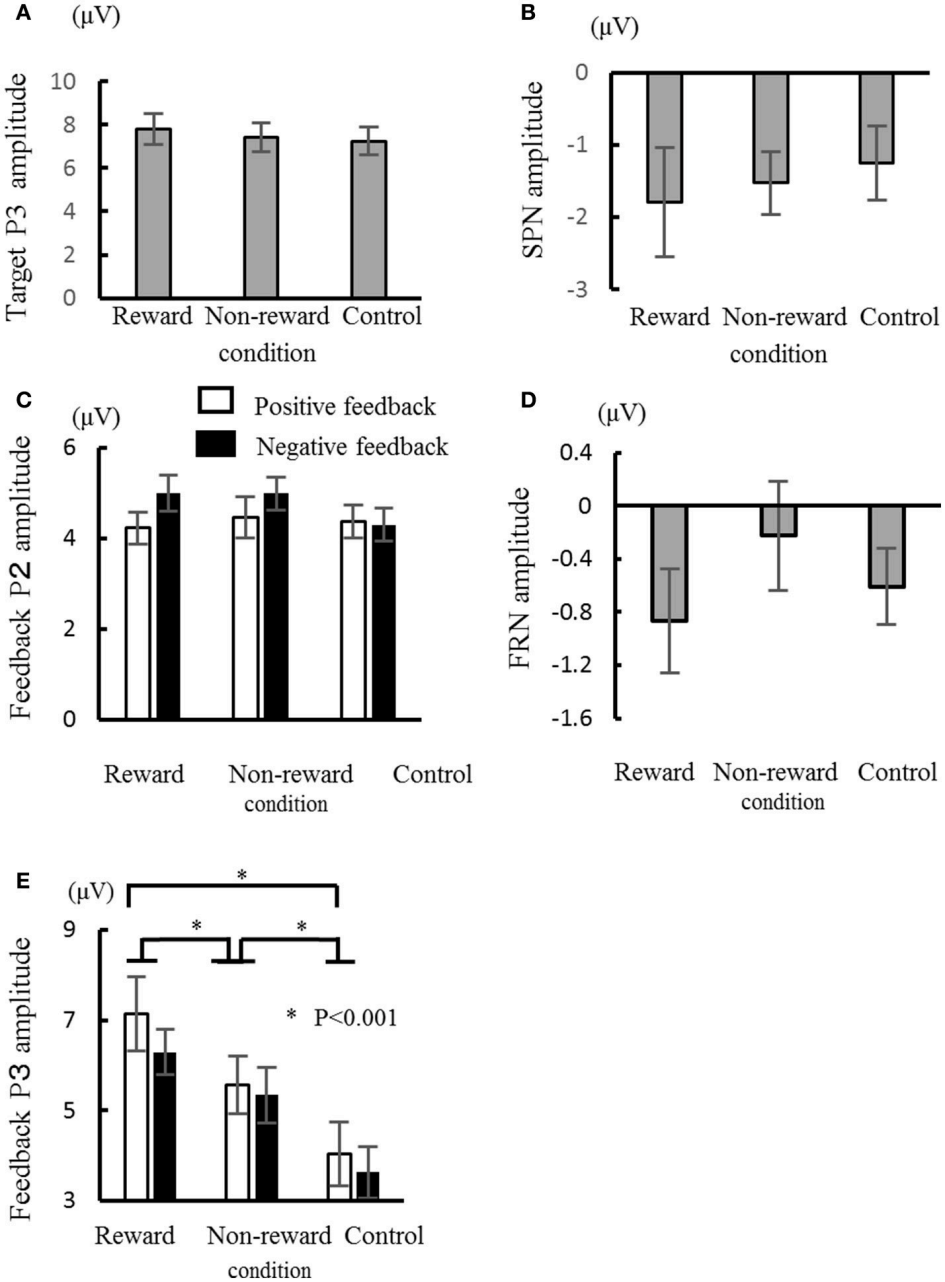
Comparisons of the ERP components in the three conditions: target P3 amplitude **(A)**, stimulus preceding negativity (SPN) amplitude **(B)**, feedback P2 amplitude **(C)**, feedback-related negativity (FRN) amplitude **(D)**, and feedback P3 amplitude **(E)**. Error bars represent the standard error. ^*^P < 0.001.

The target P2 was the largest at Cz, did not exhibit any significant main effects or interaction in terms of their amplitude and latency (*F*s < 2.4, for P2). The peak amplitude of target P3, which was largest at Cz and Pz, was mainly affected by the condition [*F*_(2, 26)_ = 4.79, ε = 0.75, *p* = 0.03, d = 0.27], where the amplitude for the reward condition was larger than that for the control condition [*p* = 0.04, Figure 3A). Similar to the amplitude, the latency was also mainly affected significantly by the condition [*F*_(2, 26)_ = 4.7, ε = 0.84, *p* = 0.024, d = 0.27], where the latency was shorter for the reward condition than the control condition (*p* = 0.007). The mean amplitude of SPN was not significantly influenced by the main effects or interactions (*F*s < 2.0, Figure 3B).

The ERPs for the feedback stimulus were analyzed using three-way ANOVA. The feedback P2 had a significant main effect of channel [*F*_(2, 26)_ = 5.54, ε = 0.64, *p* = 0.01], where the amplitude was largest at Cz (Figure 3C). Regarding for the FRN, there were neither main effects nor interactions (*F*s < 1.3, Figure 3D). The feedback P3 had a significant main effect of condition [*F*_(2, 26)_ = 52.9, ε = 0.84, *p* < 0.001, d = 0.80], where the largest amplitude was in the reward condition and the smallest amplitude in the control condition (*p*s < 0.05, Figure 3E). The interactions between channel × valence/condition were also significant (*F*s > 3.45, ε = 0.70/0.87, *ps* < 0.04, ds > 0.21). The *post-hoc* test showed that the P3 amplitude at Fz was larger for the negative feedback compared with the positive feedback (*p* < 0.05). Feedback P3 amplitude was the largest at Pz in the reward condition. There was a main effect of valence on latency, where the P3 peak latency was shorter for the positive feedback than the negative feedback [*F*_(1, 13)_ = 6.07, *p* = 0.03, d = 0.32].

**Figure 4:**
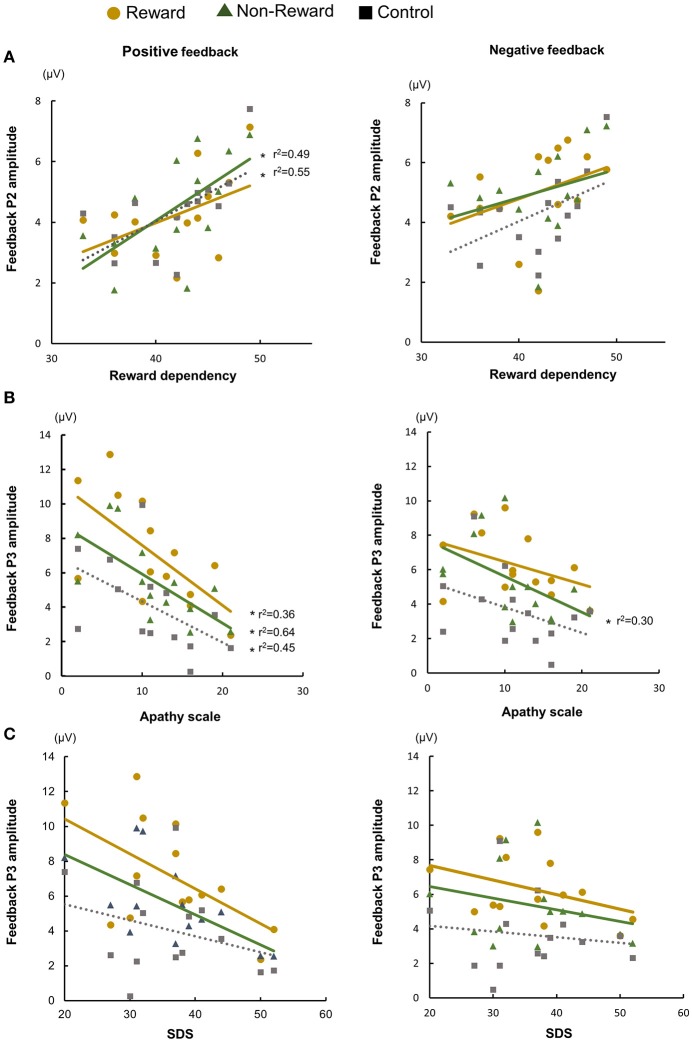
Relationship between temperaments/individual affective state and ERP amplitude: feedback P2 and reward dependency **(A)**, feedback P3 and apathy scale **(B)**, and feedback P3 and Zung's self-rating depression (SDS) scale **(C)**.

Next, we examined the correlations between the ERP components, and the individual psychological and affective characteristics (Figure 4, Supplementary Tables 1, 2). To reduce the flood of information about ERP measures in supplementary table, we averaged ERP measures across three conditions. Target P3 amplitude showed negative correlation with reaction time (*p*s < 0.05). The P2 amplitude at Cz had positive correlations with reward dependence for negative feedback in the reward condition, for positive feedback in the non-reward condition, and for positive feedback in the control condition (*p*s < 0.05). Furthermore, the P3 amplitude at Pz had negative correlations with apathy scale for positive feedback in the reward condition, for positive and negative feedback in the non-reward condition, and for positive feedback in the control condition (*p*s < 0.02).

## Discussion

In this study, we examined whether ERP components can be employed as objective measures of apathy and motivation by using a newly developed number discrimination task with or without rewards. According to the behavioral analysis, the reaction time to targets was faster in the reward condition than in the non-reward and control conditions, thereby indicating that the participants were relatively motivated by the monetary reward. We found larger ERP components for the target and feedback stimuli in the reward condition compared with other conditions, which suggests that increased neural activities are associated with enhanced motivation.

We demonstrated that the feedback P2 amplitude was positively correlated with reward dependence, and the feedback P3 amplitude was negatively correlated with the apathy score. These results imply that the feedback P2 and P3 reflected the motivation. Other ERP components, i.e., SPN and FRN, had no significant relationships with the motivational measures.

The feedback P2 was clearly elicited in all conditions in this study. P2 is considered to be a stimulus-dependent component related to an early stage of information processing (Portella et al., 2012). Potts et al. reported that the frontal P2 was the largest when the reward was unpredictable and the generator was medial frontal cortex associated with reward system (Potts et al., 2006). This evidence indicates that a larger P2 is often observed when attention is preferentially allocated to a particular stimulus, such as an imperative stimulus or performance feedback (Lackner et al., 2014). In this study, feedback P2 was correlated with reward dependence. Our result suggests that P2 amplitude increases through higher attention based on higher reward dependence. Moreover, close relationships between affective state/personality trait and the P2 component has also been reported. Regarding affective state, higher P2 amplitude was seen in shy adolescents, in individuals with anxiety disorder, and individuals with depression (Kemp et al., 2010; Han et al., 2014; Lackner et al., 2014). Affective state influenced higher attention and is explained by attention bias (Han et al., 2014) and disruption of selective attention (Kemp et al., 2010). In this study, depressive state was not associated with P2. This may be because the task does not cause affective process markedly and the degree of depressive state was mild. On the other hand, there are several studies regarding the association of reward system and reward dependence. Reward dependence was correlated with gray matter volumes in the caudate nucleus (Iidaka et al., 2006), orbitofrontal cortex, and temporal lobe (Van Schuerbeek et al., 2011); BOLD activity of substantia nigra/ventral tegmental area (Krebs et al., 2009); and opioid receptor availability in striatum and nucleus accumbens (Schreckenberger et al., 2008). These results indicate that reward dependence is associated with the reward system based on the fronto–striatal circuit. The fronto–striatal circuit may modulate P2 activity via attentional deployment.

In addition, we examined whether the P3 component is modulated by individual temperament and affective state. We found a negative correlation between the feedback P3 amplitude and score of apathy scale. P3 component is usually separated into P3a and P3b (Snyder and Hillyard, 1976). P3a is elicited by novelty or salient stimuli, for example, in an oddball task (Courchesne et al., 1975; Knight, 1984), and distributed over the fronto-central area (Conroy and Polich, 2007), suggesting its association with the frontal attention system. P3b is elicited by target stimuli in an oddball task. This component is generated partly from temporo–parietal junction (Conroy and Polich, 2007) and relates to attention and memory processing. P3 seen in a gambling task is related to motivational salience in feedback processing (Nieuwenhuis et al., 2005; Yeung et al., 2005). The feedback P3 amplitude changes depending on reward expectancy and size and the feedback value (Wu and Zhou, 2009). We consider target and feedback P3 as P3b because of the task demands and the topography. Target P3 is associated with target evaluation, feedback anticipation, and encoding contextual valence. On the other hand, feedback P3 is enhanced for the outcome with large value compared to small value and is involved in the late stage of outcome processing for motivational salience rather than contextual valence (Zheng et al., 2017). In our study, target P3 amplitude was increased in reward condition and showed correlation with reaction time but was not correlated with temperament or affective state. Referring to the study of Zheng et al. feedback P3 is related to outcome evaluation for motivational salience, and our results support their notion. We speculate that feedback P3 could be a physiological marker as motivational state.

Several studies have investigated the association between emotion/affection and the P3 component, where they demonstrated that the P3 amplitude decreased in individuals with anhedonia (Dubal et al., 2000) and depression (Foti and Hajcak, 2009; Mathis et al., 2014), which are often accompanied by apathy. In our study, we found no significant correlation between depressive state and the P3 amplitude, thereby suggesting that the P3 component may reflect apathy more directly rather than depression. Similar results were obtained for Parkinson's disease (Mathis et al., 2014), Alzheimer's disease (Daffner et al., 2001), and head trauma (Daffner et al., 2000), where these studies measured the ERP using a visual or auditory oddball task. The P3a arising mainly from the prefrontal area was also correlated with apathy in subcortical stroke patients (Yamagata et al., 2004).

Previous studies have suggested that the SPN and FRN are associated with reward expectation (Bocker et al., 1994; Pfabigan et al., 2011). The SPN amplitude depends on the amount of information with an affective or motivational value carried by the feedback stimulus (Bocker et al., 1994). The FRN is sensitive to unexpected negative feedback but also to unexpected positive feedback, which suggests that the FRN reflects expectancy and the valence of feedback. However, meaningful results were not obtained in the SPN and FRN in the current study. After the participants pressed a button, they made a prediction regarding the outcome, which would have been informed by the feedback received. The probabilities of positive and negative feedback were each fixed at 50% in this study. The probability could have influenced their surprising or disappointing reaction to feedback. It is possible that no significant changes were found in the SPN and FRN because the anticipation and expectation of the outcome were attenuated by the uncertainty of the feedback stimuli.

There was some limitations in our study. Firstly, it was conducted with healthy volunteers; therefore, the degree of apathy was mild even if they were apathetic. Severe apathy is characterized by decreased mental or behavioral reactions; therefore, although the current task was simple and easy to perform, a task that requires responses might not be suitable for studying severe apathy. Thus, we cannot be certain that similar results would be obtained in subjects with severe apathy. Secondly, the number of participants was not adequate for the correlation analysis between subjective measures and ERPs. High reliability for TCI was obtained in the English (Cloninger et al., 1994) and Japanese versions (Takeuchi et al., 2011). We also confirmed the reliability and validity of the apathy scale (Okada et al., 1998). Moreover, the stability of P2 and P3 (Thigpen et al., 2017) is known and high correlation was reported in the test-retest (McEvoy et al., 2000; Williams et al., 2005). Therefore, because there is robustness in these indicators, the results of correlation study seem acceptable even though the number of participants is not adequate for the analysis. Thirdly, there were several correlations between temperaments and states. Therefore, it is difficult to judge whether temperaments affect ERP components or individual affective states. Further studies are necessary to validate our findings before the clinical use of this method. It is desirable to generate tasks that can evaluate intrinsic, extrinsic, and novel motivation to clarify the neural basis of motivation.

In summary, the P2 and P3 may have distinct associations with motivation, where P2 reflects attention that is modulated by motivation and P3 reflects apathy more directly. The current stimulus paradigm may be useful for investigating the brain activity associated with apathy.

## Ethics statement

This study was approved by the Ethics Committee of Shimane University, and was conducted in accordance with the Declaration of Helsinki. All participants gave written informed consent.

## Author contributions

HT, KO, and SY make substantial contributions to conception and design, and acquisition of data, and analysis and interpretation of data and also participate in drafting the article or revising it critically for important intellectual content. Every authors give final approval of the version to be submitted and any revised version. Every authors give agreement to be accountable for all aspects of the work in ensuring that questions related to the accuracy or integrity of any part of the work are appropriately investigated and resolved.

### Conflict of interest statement

The authors declare that the research was conducted in the absence of any commercial or financial relationships that could be construed as a potential conflict of interest.

## Abbreviations

ACC: anterior cingulate cortex
ANOVA: analysis of variance
EEG: electroencephalogram
ERP: event-related potentials
FRN: feedback-related negativity
SPN: stimulus preceding negativity
TCI: temperament and character inventory.
