# Supplementary material for: Do Event-Related Evoked Potentials Reflect Apathy Tendency and Motivation?
Source: Front Hum Neurosci. 2018 Jan 31;12:11. doi: 10.3389/fnhum.2018.00011 (PMC5797740; doi:10.3389/fnhum.2018.00011)
Supplement: Supplementary file 1 [file Table1.docx]

Supplementary table 1

Correlations between measures of ERP components, and psychological and affective characteristics and behavioral measures

|  | NS | HA | RD | AS | SDS | mean RT | mean RT(R) | mean RT(N) | mean RT(C) | Error rate |
| --- | --- | --- | --- | --- | --- | --- | --- | --- | --- | --- |
| TargetP2 | .432 | −.101 | .410 | −.095 | −.460 | −.042 | −.125 | −.125 | .090 | −.438 |
| TargetN2 | .344 | .187 | .340 | .366 | −.278 | −.327 | −.130 | −.451 | −.459 | .060 |
| TargetP3 | −.024 | −.331 | .128 | −.373 | .007 | −**.626^*^** | −**.556^*^** | −.477 | −**.569^*^** | .098 |
| SPN(Fz) | .377 | −.258 | **.604^*^** | −.430 | −.460 | −.490 | −.446 | −.455 | −.429 | 0.000 |
| SPN(Cz) | .260 | −.161 | **.545^*^** | −.298 | −.328 | −.446 | −.393 | −.358 | −.389 | .173 |
| SPN(Pz) | .362 | −.276 | .428 | −**.547^*^** | −**.659^*^** | −.499 | −.495 | −.508 | −.332 | .129 |
| FB P2 | .432 | −.013 | **.701^**^** | .073 | −.178 | .134 | .116 | .156 | .240 | −.420 |
| FB P2 Posi | .404 | .051 | **.589^*^** | .033 | −.018 | .248 | .253 | .240 | .292 | −**.622^*^** |
| FB P2 Nega | .412 | −.130 | **.697^**^** | −.064 | −.359 | .073 | .007 | .090 | .165 | −.356 |
| FRN | .086 | .101 | .280 | .082 | .163 | −.455 | −.433 | −.371 | −.336 | .013 |
| FB P3 | .035 | −.322 | .201 | −**.647^*^** | −.231 | −.521 | −.495 | −.407 | −.433 | .098 |
| FB P3 Posi | −.049 | −.238 | .190 | −**.583^*^** | −.315 | −.446 | −.446 | −.332 | −.358 | .124 |
| FB P3 Nega | −.079 | −.267 | .082 | −**.598^*^** | −.130 | −.429 | −.446 | −.279 | −.349 | .120 |

P value: *<0.05 **<0.01, NS: novelty seeking, HA: harm avoidance, RD: reward dependence, AS: apathy scale, SDS: self-rating depression scale, (R): reward condition, (N): non-reward condition, (C): control condition

Supplementary table 2

Correlations between individual psychological and affective characteristics and behavioral measures during the discrimination task

|  | NS | HA | RD | AS | SDS | mean RT | mean RT(R) | mean RT(N) | mean RT(C) | Error rate |
| --- | --- | --- | --- | --- | --- | --- | --- | --- | --- | --- |
| NS | 1 | −**.584^*^** | .458 | −.429 | −.406 | −.241 | −.263 | −.359 | −.033 | −.083 |
| HA | −**.584^*^** | 1 | −.150 | **.669^**^** | **.564^*^** | .119 | .114 | .219 | −.005 | .163 |
| RD | .458 | −.150 | 1 | −.254 | −**.555^*^** | −.079 | −.096 | −.147 | .032 | −.299 |
| AS | −.429 | **.669^**^** | −.254 | 1 | **.640^*^** | .274 | .384 | .326 | .013 | .026 |
| SDS | −.406 | **.564^*^** | −**.555^*^** | **.640^*^** | 1 | .225 | .207 | .357 | .060 | .035 |
| mean RT | −.241 | .119 | −.079 | .274 | .225 | 1 | **.913^**^** | **.966^**^** | **.890^**^** | −**.666^**^** |
| mean RT(R) | −.263 | .114 | −.096 | .384 | .207 | **.913^**^** | 1 | **.832^**^** | **.650^*^** | −**.623^*^** |
| mean RT(N) | −.359 | .219 | −.147 | .326 | .357 | **.966^**^** | **.832^**^** | 1 | **.858^**^** | −**.611^*^** |
| mean RT(C) | −.033 | -.005 | .032 | .013 | .060 | **.890^**^** | **.650^*^** | **.858^**^** | 1 | −**.605^*^** |
| Error rate | −.083 | .163 | −.299 | .026 | .035 | −**.666^**^** | −**.623^*^** | −.611^*^ | −**.605^*^** | 1 |

P value: *<0.05 **<0.01, NS: novelty seeking, HA: harm avoidance, RD: reward dependence, AS: apathy scale, SDS: self-rating depression scale, (R): reward condition, (N): non-reward condition, (C): control condition
